# Design, synthesis and characterization of linear unnatural amino acids for skin moisturization

**DOI:** 10.1111/ics.12351

**Published:** 2016-07-24

**Authors:** N. R. Arezki, A. C. Williams, A. J. A. Cobb, M. B. Brown

**Affiliations:** ^1^ Department of PharmacyUniversity of ReadingPO Box 226WhiteknightsReadingRG6 6AP; ^2^ MedPharm Ltd.50 Occam RoadSurrey Buisness ParkGuildford, GU2 7ABUK

**Keywords:** chemical synthesis, deliquescence relative humidity, delivery, hygroscopic, natural moisturizing factor, skin barrier

## Abstract

**Objectives:**

This work aimed to design, synthesize and characterize replacement natural moisturizing factor (NMF) composed of modified hygroscopic linear amino acids to pre‐empt or repair skin barrier dysfunction.

**Methods:**

Following synthesis and characterization, thermo‐gravimetric analysis and quantum mechanics molecular modelling quantified and depicted water binding to the new compounds. Deliquescence relative humidity demonstrated the water‐scavenging ability of the compounds, whereas snake skin moisturizing studies showed they increased water uptake into snake skin.

**Results:**

From thermal analysis, *N*‐hydroxyglycine showed greatest water‐holding capacity followed by *N*‐hydroxyserine, l‐homoserine and *α*‐hydroxyglycine; coupled with quantum mechanics molecular modelling, between 8 and 12 molecules of water could associate with each molecule of either *N*‐hydroxyglycine, *N*‐hydroxyserine or l‐homoserine. All of our modified amino acids were efficacious and induced similar or greater water uptake compared with the established moisturizing compounds hyaluronic acid, glycerine and urea in snake skin. Incorporated at 10% in Oilatum, *N*‐hydroxyserine induced >200% greater moisture uptake into dry snake skin compared to treatment with water alone, with efficacy related to the molecule structure and ability to bind to 12 water molecules. Oilatum cream spiked with all our unnatural amino acid hydrotropes increased water uptake into snake skin compared with Oilatum alone.

The compound series was designed to elucidate some structure – efficacy relationships. Amino acid chirality did not affect the water‐holding capacity but did affect uptake into skin. Compounds with high melting points and bond energies tended to decrease water‐holding capacity. With isosteric replacement, the more electronegative atoms gave greater water‐holding capacities.

**Conclusions:**

This work demonstrates the potential of unnatural amino acid hydrotropes as skin moisturizers and has developed some predictive ‘rules’ for further design and refinement of chemical structures.

## Résumé

**Objectifs:**

Ce travail vise à concevoir, synthétiser et 0 caractériser le remplacement du Natural Moisturising Factor (NMF) composé d'acides aminés linéaires hygroscopiques modifiés pour anticiper ou réparer un dysfonctionnement de la barrière cutanée.

**Methode:**

Après la synthèse et la caractérisation, l'analyse thermo‐gravimétrique et la modélisation par la mécanique quantique moléculaire permettent de quantifier et représenter les nouveaux composés liés à l'eau. A humidité relative on démontre la capacité des composés piégeant l'eau, tandis que les études d'hydratation de la peau de serpent ont montré qu'ils ont augmenté l'absorption de l'eau dans la peau de serpent.

**Résultats:**

De l'analyse thermique, la N‐hydroxyglycine a montré la plus grande capacité de rétention d'eau suivie de N‐hydroxyserine, l‐homosérine et α‐hydroxyglycine; couplée avec la modélisation mécanique quantique moléculaire, entre 8 et 12 molécules d'eau pourrait s'associer à chaque molécule de l'une des N‐hydroxyglycine, N‐hydroxyserine ou lL‐homosérine. Tous les acides aminés modifiés sont efficaces et conduisent à une absorbance d'eau similaire ou supérieure par rapport aux composés hydratants établis tels que l'acide hyaluronique, la glycérine et l'urée dans la peau de serpent. Incorporée à 10% dans Oilatum, la N‐hydroxyserine induit une plus grande absorption de l'humidité dans la peau de serpent sèche de 200% par rapport au traitement avec de l'eau seule, avec une efficacité liée à la structure des molécules et la capacité de se lier à 12 molécules d'eau. Une crème Oilatum renforcée avec tous nos hydrotropes d'acides aminés non naturels augmente l'absorption d'eau dans la peau de serpent par rapport à Oilatum seul.

La série des composés a été conçue pour élucider certaines relations structure ‐ efficacité. La chiralité des acides aminés n'a pas affecté la capacité de rétention d'eau, mais a affecté l'absorption dans la peau. Les composés ayant des points de fusion et des énergies de liaison ont tendance à diminuer la capacité de rétention d'eau. Avec le remplacement isostérique, les atomes plus électronégatifs ont donné une plus grande capacité de rétention d'eau.

**Conclusions:**

Ce travail démontre le potentiel des acides aminés non naturels hydrotropes comme hydratants pour la peau et a développé des «règles» de prédiction pour poursuivre la conception et l'amélioration des structures chimiques.

## Introduction

Optimal water content in human skin is essential to maintain the excellent barrier properties of this tissue 1. It is the outermost layer of this multilayered structure, the stratum corneum, which regulates water loss from the body and acts as a barrier to exogenous chemical ingress 1. Underlying the stratum corneum, filaggrin is a protein synthesized in the viable epidermis which is subsequently hydrolysed to generate a complex mixture of hygroscopic free amino acids, amino acid derivatives and salts which together constitute the natural moisturizing factor (NMF) and which is largely responsible for maintaining the water content of the stratum corneum 2, 3. Filaggrin gene mutations with consequent reductions in tissue NMF levels have been shown in patients with ‘dry skin’ and conditions such as atopic dermatitis 4. Natural moisturizing factor constituents include sodium pyrrolidone carboxylic acid (PCA), lactic acid and urea, all of which are highly water‐soluble and hygroscopic making them efficient humectants 5 and each comprise approximately 8–10% of NMF 6. Inorganic ions account for 5% of NMF including potassium, sodium, magnesium and calcium 7. The largest component (~40%) of NMF is the free amino acids 7. Of these, l‐serine is the most abundant (~36%) followed by glycine (22%) and l‐alanine (13%) 8. Histidine, ornithine, citrulline and arginine all account for 6–8% of free amino acids within NMF 8, 9. It should be noted that there are site‐to‐site variations in both relative and absolute amino acid contents in NMF with typically lower levels of serine and citrulline recovered from tape strips of the jaw or cheek than from the back, torso or calf 10, 11 and lower levels of NMF reported in the cheek when compared to that in the forearm 12.

The NMF amino acids are shed with skin desquamation or are recycled. Further, these water‐soluble materials can be lost during washing and bathing; Robinson *et al*. demonstrated significant loss of total amino acids from forearm skin following 10 min of soaking in 40°C water 13. Interestingly, the total amino acid content rapidly recovered (within 4 h) in the deeper layers of the stratum corneum but not at the superficial layers where recovery required desquamation and replacement with amino acid containing keratinocytes. In seeking a replacement NMF to manage dry skin disorders, we designed, synthesized and evaluated a series of unnatural linear amino acid hydrotropes with enhanced hygroscopicity and hydrogen‐bonding propensities. Beyond amino acids, studies of the hygroscopic properties of humic materials in soils have shown that compounds with higher oxygen‐to‐carbon ratios (O/C) typically exhibit greater hygroscopic properties 14. Thus, the unnatural amino acid hydrotropes were designed to provide a range of O/C from 0.75 to 1.5. Various linear amino acids with O/C < 1 including amino acid components of NMF (serine, glycine and alanine) were also evaluated alongside the well‐established humectant, urea. This extensive compound library was used to elucidate broad structure–activity relationships aiming to explain the chemical features that are responsible for the water retention properties of hydrotropes.

The compound series was evaluated using deliquescence relative humidity (DRH) to understand the ‘water‐scavenging’ ability of the compounds. DRH is the relative humidity at which the compound deliquesces, that is the relative humidity at which the compound absorbs so much water that it dissolves within the absorbed water. It is expected that with increasing water‐holding capacity, the DRH will decrease and so the lower the DRH, the greater the propensity that the compound has for scavenging water 15.

Thermo‐gravimetric analysis (TGA) was used to quantify water binding to the unnatural amino acids and to (positive control) established humectants such as urea. Complementing the experimental data on the number of water molecules bound per molecule of our unnatural amino acid hydrotropes, quantum mechanics molecular modelling (QMMM) was used to predict the number and binding sites of water molecules associating with each unnatural amino acid and with urea. Using the number of hydrogen bond‐to‐bound water ratio allowed assessment of whether the water binds directly to the compound or to other water molecules.

Efficacy of the moisturizers was evaluated in snake skin, selected as a mimic of ‘dry’ human skin because this tissue does not contain NMF as would be the case with excised human tissue samples 16. Snake skin was treated variously with our novel compounds, established humectants and commercial preparations (some spiked with our materials) before exposure to a controlled humidity with water uptake assessed gravimetrically.

## Materials and methods

### Materials

Reagents and solvents were purchased from Sigma–Aldrich (UK). Urea, glycine, d‐serine, l‐serine, l‐alanine, l‐threonine, l‐cysteine, l‐asparagine, l‐aspartic acid and l‐homoserine were also from Sigma–Aldrich (UK). Oilatum cream (light liquid paraffin 6.0% w/w, white soft paraffin 15.0% w/w in a cream including cetostearyl alcohol, benzyl alcohol, glycerol and purified water) and Cetraben emollient cream (light liquid paraffin 10.5% w/w, white soft paraffin 13.2% w/w in a cream including emulsifying wax, cetostearyl alcohol, glycerol and purified water) were purchased from Lloyds Pharmacy.

All reactions containing moisture‐sensitive reagents were carried out under a nitrogen atmosphere using standard line techniques. For product characterization, thin‐layer chromatography (TLC) was performed on aluminium plates coated with silica 60 F_254_. The plates were visualized using UV light, iodine and KMnO_4_. Flash chromatography used high‐grade silica gel, pore size 60 Å, 220–440 mesh, 35–37 μm. Melting points were recorded on a Stuart SMP3 melting point apparatus. IR spectra were recorded on a PerkinElmer Spectrum RX I FT‐IR spectrometer as a solution (stated) or as a solid on a PerkinElmer 1000 FT‐IR spectrometer; characteristic peaks are reported in cm^−1^. NMR spectra were recorded on a Bruker 400 MHz in the deuterated solvent stated or on a Bruker 700 MHz in the solid state. The field was locked by referencing to the relevant deuteron resonance, and all characteristic peaks are reported in ppm. Mass spectra were recorded on a Thermo Scientific low Orbitrap XL with samples loaded by a Thermo Scientific autosampler. Powder X‐ray diffraction data were collected on a Bruker D8 Advance in capillary transmission mode. Borosilicate 0.7‐mm capillaries were used for collection. Collections were made over a 24‐h period and data were scanned from 5 to 105°.

### Synthesis of modified linear amino acids

Synthetic methodologies are described below. Analytical validation of successful synthesis using the above techniques is provided in the (Table S1) to this paper.

#### Benzaldoxime


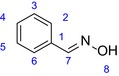


Benzaldehyde (2.00 mL, 20 mmol) was stirred in a mixture of ice: water: ethanol (2 : 1 : 1, v/v/v, 20 mL) at room temperature. Hydroxylamine hydrochloride (1.386 g, 20 mmol) was added to the stirred mixture followed by 50% aqueous sodium hydroxide (4.00 mL, 40 mmol), while keeping the temperature below 30°C. After stirring for 1 h at room temperature, the mixture was extracted with diethyl ether (2 × 25 mL). The aqueous extract was acidified to pH 6 using conc. HCl, while keeping the temperature below 30°C, before again being extracted with diethyl ether (2 × 25 mL). The combined organic extracts were dried over MgSO_4_, filtered and concentrated *in vacuo* to yield a colourless liquid (2.425 g, 100%, 20 mmol).

#### 2‐bromo‐3‐hydroxypropanoic acid


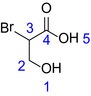


Potassium bromide (350.6 g, 2.94 mol) and l‐serine (100.0 g, 0.95 mol) were stirred in 2.5 M aqueous sulphuric acid (1.8 L) at 0°C. Sodium nitrite (92.00 g, 1.33 mol) was added slowly to ensure the temperature remained below 5°C. Half the sodium nitrite was added over the first 8 h; the mixture was left stirring overnight before the remaining sodium nitrite was added over the next 30 min. After stirring for 2.5 h at 0°C, the mixture was extracted with diethyl ether (3 × 250 mL). The organic extracts were combined, dried over MgSO_4_, filtered and concentrated *in vacuo*. The residue was then further concentrated *in vacuo* over 8 h to yield the title product (154.7 g, 96%, 0.915 mol).

#### Ethyl 2‐bromo‐3‐hydroxypropanoate


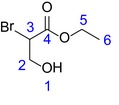


Concentrated sulphuric acid (2.8 mL, 0.003 mL per mmol) was added slowly to a stirred mixture of 2‐bromo‐3‐hydroxypropanoic acid (154.7 g, 0.92 mol) in absolute ethanol (1.83 L, 2 mL per mmol) before heating under reflux for 1.5 h. The mixture was cooled, and ice‐cold water (1.83 L) was added and extracted with diethyl ether (2 × 1.8 mL). The organic extracts were combined and washed with ice‐cold water (1.8 L), 5 M aqueous sodium carbonate (2 × 1.8 L) and saturated brine (1.8 L). The organic extract was dried over magnesium sulphate, filtered and concentrated *in vacuo* to yield the title product (179.3 g, 99.5%, 0.91 mol).

#### *N*‐hydroxyglycine


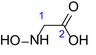


Sodium (0.4626 g, 0.02 mol) was added to a stirred mixture of benzaldoxime (2.400 g, 0.02 mol) in absolute ethanol (40 mL). Ethyl bromoacetate (2.44 mL, 0.022 mol, 1.1 equiv.) was added, and the mixture was stirred until the pH reached 7 which took 3 h. The mixture was filtered, and the solid was washed with chloroform (2 × 40 mL). The combined filtrate was concentrated *in vacuo*. The residue was taken up in diethyl ether (50 mL) and placed at 4°C overnight. The mixture was filtered and washed with cold diethyl ether, and the solid was dried under suction (3.158 g). The nitrone (1.5 g) solid was stirred in conc. HCl (20 mL) and heated under reflux for 0.5 h. The mixture was concentrated *in vacuo*. The residue was taken up into water, and the pH was raised to 6 using ammonium hydroxide solution. The mixture was cooled at 4°C for 48 h and filtered and the solid was recrystallized from hot aqueous ethanol (75%) to yield (0.8095 g, 8.90 mmol, 76%).

#### *N*‐hydroxyserine


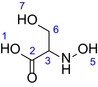


Sodium (0.4600 g, 0.02 mol) was added to a stirred mixture of benzaldoxime (2.420 g, 0.02 mol) in absolute ethanol (40 mL). Ethyl 2‐bromo‐3‐hydroxypropanoate (4.334 g, 0.022 mol, 1.1 equiv.) was added, and the mixture was stirred until the pH reached 7 which took 3 h. The mixture was filtered, and the solid was washed with chloroform (2 × 40 mL). The combined filtrate was concentrated *in vacuo*. The residue was taken up into diethyl ether (50 mL) and placed at 4°C overnight. The mixture was filtered and washed with cold diethyl ether and the solid was dried under suction (2.413 g). The nitrone (1.5 g) solid was stirred in conc. HCl (20 mL) and heated under reflux for 0.5 h. The mixture was concentrated *in vacuo*. The residue was taken up into water (10 mL), and the pH was raised to 6 using ammonium hydroxide solution. The mixture was cooled at 4°C for 48 h and filtered and the solid was recrystallized from hot aqueous ethanol (75%). Acetone was added to encourage the product to drop out of solution to yield the title product (0.6724 g, 5.56 mmol, 88%).

#### *α*‐hydroxyglycine


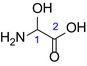


Ammonium acetate (9.473 g, 0.1 mol, 2 equiv.) in ice‐cold water (10 mL) was added to a stirred solution of glyoxylic acid monohydrate (4.602 g, 0.05 mol, 1 equiv.) in ice‐cold water (10 mL). A white precipitate appeared within minutes of stirring. After 45 min of stirring and 2 h of standing at 0°C, the reaction mixture was filtered. The white precipitate was washed with water (20 mL) and methanol (2 × 10 mL) to yield the crude title product (4.695 g, 0.051 mol, 103%). The crude product (4.170 g) was dried by a high vacuum pump for 5 h to afford the title product (4.0571 g, 97%).

### Deliquescence relative humidity

Triplicate vials containing 1 mL of water were saturated with the amino acids. The vials were individually placed in a desiccator at 32°C (which had also been stored at 32°C for 48 h prior to testing) and the desiccator lid clamped to ensure a good seal. The temperature and percentage relative humidity shown on the thermo‐hygrometer within the desiccator (K‐type digital hand‐held, ATP, Leicestershire, UK) were recorded at 0, 0.25, 0.5, 1, 2, 3, 4, 5 and 24 h.

Zinc nitrate and water alone were employed as controls and to validate the system; water provided the expected 100% DRH value whereas a saturated solution of zinc nitrate recorded a DRH of 45.8% at 32°C (literature states that a saturated solution of zinc nitrate should give a RH of 42% at 20°C) 17. All values are means ± SD from triplicate experiments.

### Thermo‐gravimetric analysis (TGA)

Thermo‐gravimetric analysis was performed on a TGA Q50 TA instrument, UK. All samples were lightly ground to disperse coarse aggregates using a pestle and mortar (unless otherwise stated) and dried by storing in a vacuum desiccator over silica for 1 week. The dried samples were then placed into controlled relative humidity chambers at room temperature for 1 week before TGA analysis. The relative humidity (RH) of the desiccators was as follows: 0% RH was achieved using a vacuum desiccator over silica, 33% RH was achieved using a desiccator containing a saturated solution of zinc nitrate, 40% RH was achieved using a desiccator containing a saturated solution of potassium carbonate and 100% RH was achieved using a desiccator containing water. It should be noted that saturated zinc nitrate gave an initial RH of 42% at 20°C 17, but this equilibrated at the recorded 33% RH due to the presence of the hygroscopic unnatural amino acids. Samples were heated in the instrument from 25°C to 250°C at 10°C min^−1^. Mass loss was recorded over the temperature ramp from which the water content could be calculated as a mole ratio according to, for example:

l‐homoserine stored at 100% RH, weight loss on heating 53.3% (assumed water).

Water molecular weight 18; l‐homoserine molecular weight 119.

Moles water = 53.3/18 = 2.96 mol; moles l‐homoserine = (100–53.3)/119 = 0.39 moles.

Mole ratio = 2.96/0.39 = 7.59, so 7.59 molecules water per molecule of l‐homoserine.

Prior to evaluating the water content in snake skin, the approach was validated by heating a sample of *α*‐lactose monohydrate which lost 5% of its weight (assumed water) over the temperature scan, equating to one molecule of water per lactose molecule (mw *α*‐lactose monohydrate is 360).

### Quantum mechanics molecular modelling (QMMM)

Energy minimizations were performed on l‐homoserine, *α*‐hydroxyglycine, *N*‐hydroxyglycine, *N*‐hydroxyserine and urea; structures were drawn into Gmolden to obtain the Cartesian coordinates before Gaussian energy minimization was performed on each structure. The resultant lowest energy structures were then used for the addition of water molecules. Water molecules were added sequentially and positioned such that each water molecule bound to a hydrogen bond donor or acceptor, with each bound water being positioned in various orientations. The structure with bound water then had a Gaussian energy minimization performed on it, with the structure providing the lowest energy used to add the subsequent water molecule. All the unnatural amino acids (and urea) were modelled until 12 water molecules per one compound molecule had been run.

### Snake skin moisturizing study

Shed snake skin (*Pantherophis guttatus*, corn snake; collected from local herpetologists and stored dry) was used to assess the moisturizing properties of the unnatural linear amino acid hydrotropes alongside natural amino acids found in NMF; established moisturizers such as urea and hyaluronic acid were tested and the novel materials were also incorporated into Oilatum cream, a commercial emollient widely used in dry skin disorders. Compounds were added at 10% w/w in aqueous solutions or the cream, glycerine was used as a neat liquid (positive control), and hyaluronic acid was additionally used as a 1% w/w aqueous solution. In parallel, samples were treated with water as a control against which the weight increase due to hydrotrope treatment was assessed. All systems and tests were in triplicate.

Snake skin, from the dorsal side of the same donor, was cut into 1 cm^2^ samples. Each sample was weighed (Sartorius CPA225D; to 0.01 mg) and placed directly onto ~1 mL (or 100 mg) of a test formulation (or water control) in a glass vial; membranes were carefully floated onto or placed on formulations to avoid air bubbles between the liquid and the skin before vials were sealed and stored at 32°C. After 24 h, the snake skin was removed, blotted dry on filter paper, removing surface residual compound, and weighed. The samples were transferred into individual open Petri dishes before being placed over silica in a vacuum desiccator at 0% relative humidity (RH) for 48 h before being reweighed. The Petri dishes with the skin samples were then transferred into a desiccator at a 70% RH at 32°C (controlled by a 1 : 1 NaCl: Na_2_CO_3_ saturated solution). After 24 h, the snake skin was removed from the desiccator and weighed. The weight of the skin when placed in the desiccator at 70% RH (i.e. after hydrotrope treatment) and the weight after being removed from the 70% RH desiccator were used to calculate the percentage weight increase, assumed to be water.

### Statistical analysis

Data are presented as mean ± SD, and statistical analyses were performed in Excel using one‐way ANOVA with significance defined as *P* < 0.05.

## Results

The unnatural linear amino acids were synthesized by methods modified from the literature; *α*‐hydroxyglycine was synthesized with a yield of 97% by the method described by Hoefnagel 18. The X‐ray powder diffraction pattern on a zero background at room temperature and 0% RH confirmed that *α*‐hydroxyglycine was successfully synthesized and showed the packing to be the non‐centrosymmetric Pna2_1_ space group (see Figure S1). *N*‐hydroxyglycine was successfully synthesized with a good yield of 76% by the method described by Buehler 19. A melting point of 137–139°C was observed, which is in agreement with the literature value of 138–139°C 20. *N*‐hydroxyserine was successfully synthesized based on the method described by Buehler 19.

Following synthesis and characterization of our unnatural amino acid hydrotropes, these compounds were investigated alongside natural amino acid constituents of NMF and commercially used humectants by deliquescence relative humidity. The results for the compounds are given in Table 1 along with their O/C ratio and melting point.

**Table 1:** Compounds with their structures, deliquescence relative humidity (DRH), oxygen‐to‐carbon (O/C) ratio and melting point. The compounds emboldened are the unnatural linear amino acids synthesized here as replacement natural moisturizing factor. Non‐emboldened materials are established moisturizers and natural amino acid components of NMF

Compound	Structure	DRH (%)	O/C ratio	Melting point (°C)
***N*‐hydroxyglycine**	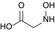	68.1 ± 3.0	1.5	137–139
***N*‐hydroxyserine**	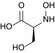	68.3 ± 2.1	1.3	159–163
**l‐homoserine**	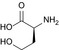	71.4 ± 4.7	0.75	203 (Dec)
Urea	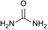	75.0 ± 6.0	1	132–133
Glycine	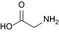	79.1 ± 0.2	1	240 (Dec)
***α*‐hydroxyglycine**	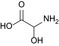	79.7 ± 2.3	1.5	105–108 (Dec)
d‐serine	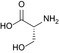	82.1 ± 8.8	1	201–203 (Dec)
l‐serine	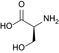	82.5 ± 3.7	1	200–203 (Dec)
l‐alanine	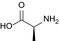	83.1 ± 11.0	0.67	297 (Dec)
l‐threonine	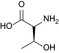	86.0 ± 8.9	0.75	256 (Dec)
l‐cysteine	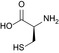	89.6 ± 13.1	0.67	240 (Dec)
l‐asparagine	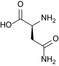	91.2 ± 10.7	0.75	234–235
l‐aspartic acid	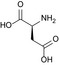	100 ± 0.0	1	270–271

Mean (± SD, *n* = 3) deliquescence relative humidity recorded for the compounds tested; the unnatural amino acids are emboldened with other hydrotropes selected to develop structure–activity relationships. Dec = decomposed.

**Figure 1:**
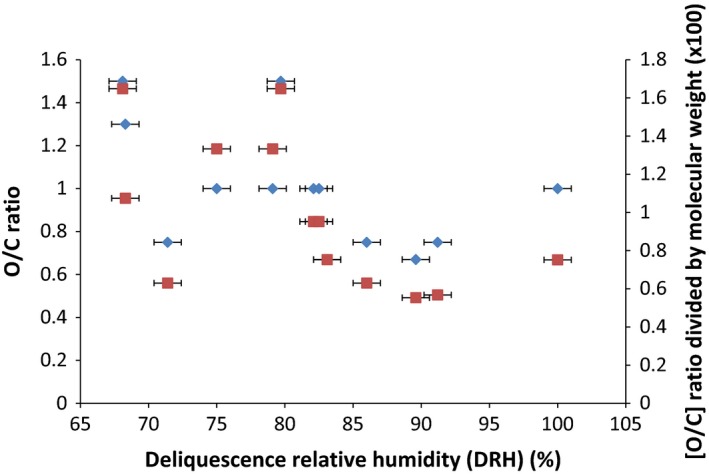
A graph showing the oxygen‐to‐carbon (O/C) ratio for a compound plotted against the deliquescence relative humidity (DRH) measured experimentally for that compound and the oxygen‐to‐carbon (O/C) ratio for a compound divided by its molecular weight plotted against the deliquescence relative humidity (DRH) measured experimentally for that compound. The O/C ratio data points are shown as diamonds and [O/C]/Mw ratio data points illustrated as squares.

From the background literature, a correlation had been anticipated between the O/C ratio of the compounds and their deliquescence relative humidity; Fig. 1 plots these data alongside the molecular weight correction for the O/C ratio against DRH.

Following storage at 100% RH, compounds were studied by thermo‐gravimetric analysis with weight loss assumed to be that of water associated with the hydrotropes. Except for *N*‐hydroxyglycine (where TGA did not discriminate between ‘bound’ and ‘outer hydration shell’ water, discussed below), sequential modelling of water addition to the hydrotropes broadly agreed with the experimental thermal data as shown in Table 2.

**Table 2:** Compounds with the number of bound water molecules calculated from thermo‐gravimetric analysis (TGA). The unnatural amino acids and urea also show the number of bound water molecules determined from quantum mechanics molecular modelling (QMMM) to illustrate that the experimental data are supported by the theoretical calculations for water‐holding capacity

Compound	Number of water molecules per compound molecule calculated from 100% RH TGA data	Number of water molecules per compound molecule calculated from quantum mechanics molecular modelling
l‐homoserine	7.5 ± 0.3	8
*α*‐hydroxyglycine	1.7 ± 0.1	3
d‐serine	0.5 ± 0.2	–
l‐serine	0.4 ± 0.2	–
*N*‐hydroxyglycine	23.6 ± 3.6	8
*N*‐hydroxyserine	11.5 ± 0.5	12
Urea	5.0 ± 0.1	5

Mean (± SD, *n* = 3) number of water molecules calculated from TGA experiments recorded for the compounds tested.

**Figure 2:**
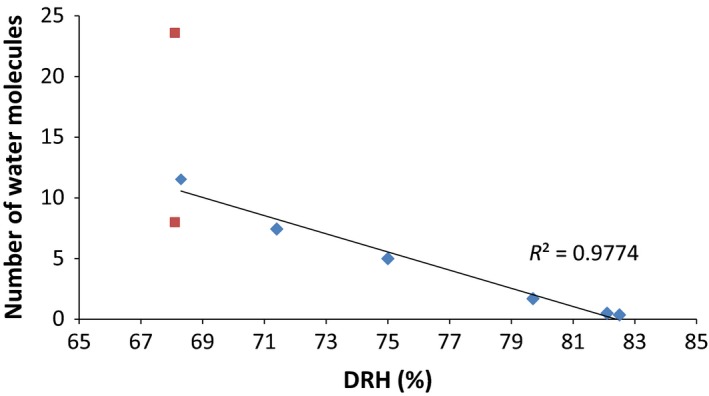
A graph showing the number of water molecules per molecule of linear amino acid, calculated from thermo‐gravimetric analysis (TGA) after the compounds had been stored at 100% relative humidity for 1 week prior to testing, against the deliquescence relative humidity (DRH) for that compound. Squares represent the data for *N*‐hydroxyglycine; TGA analysis suggested 24 water molecules associated with each *N*‐hydroxyglycine molecule, but ‘bound’ and ‘outer hydration shell’ water could not be distinguished from these data. Quantum mechanics molecular modelling showed that 8 water molecules directly bind to each molecule of *N*‐hydroxyglycine which, when plotted against DRH, shows concordance with the data from the other hydrotropes.

To cross‐validate the use of deliquescence relative humidity as a measure of water‐holding capacity of the molecules, the experimentally determined number of water molecules associated with each compound (Table 2) was plotted against their DRH values (from Table 1). This correlation is shown in Fig. 2 with the square symbols relating to the anomalous results for *N*‐hydroxyglycine.

**Figure 3:**
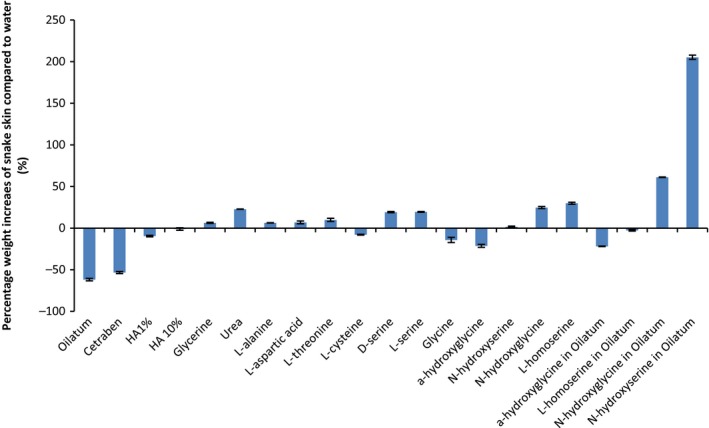
A bar chart showing the percentage weight increase of snake skin after storage at 32°C and 70% RH compared with weight increase after treatment with water (control), following 24‐h treatment with the compound. Mean ± SD,* n* = 3.

The ability of the hydrotropes to retain water in a biological tissue was investigated. Following treatment, the skin samples were blotted, dried and weighed. They were then exposed to 70% RH for 24 h before reweighing; the mass changes, assumed due to water, are in Fig. 3.

## Discussion

### Deliquescence relative humidity (DRH)

In ‘dry skin’ conditions, including atopic dermatitis, reduced levels of NMF are associated with barrier dysfunction; therefore, replacing this natural hygroscopic mixture could pre‐empt stratum corneum barrier disturbance or support its repair. The design and synthesis of the novel unnatural amino acids exploited literature reports on humic materials in soils which showed that compounds with higher oxygen‐to‐carbon ratios (O/C) typically exhibit greater hygroscopic properties 14. The compounds were thus constructed to explore the effects of homologation, increased or decreased O/C ratio, and chirality on hygroscopicity. There are currently no reports on the effects of changing the homology or the chirality of compounds on their hygroscopic properties. As serine and glycine are the most abundant amino acid in NMF, our structures were designed from these starting materials so that l‐serine and glycine could be used as a control. Other NMF components were also used when measuring hygroscopicity and water retention abilities by moisture sorption, thermo‐gravimetric analysis and molecular spectroscopy to allow some broad structure–activity relationships to be developed.

Compared with the natural amino acids, all four of the new unnatural amino acids demonstrated lower deliquescence relative humidities and consequently greater water‐holding capacities. Indeed, three of the new hydrotropes had greater water‐holding tendencies than the well‐established humectants glycine and urea. Urea and glycine are both widely used in cosmetic and pharmaceutical preparations for skin moisturization and in barrier preparations as aids to skin repair 2, 21, 22.

*N*‐hydroxyglycine and *N*‐hydroxyserine gave the lowest DRH values of our amino acids. Both compounds possess an O/C ratio greater than 1 and had DRH values below that of glycine and urea, both of which have an O/C ratio of 1, indicating that the O/C ratio has some bearing on the water‐holding capacity of a compound.

It was thus expected that increasing the O/C ratio would raise the water‐holding capacity of the compound, which in turn would decrease the DRH; a plot of O/C ratio against DRH is given in Fig. 1. Unexpectedly, the data showed no such correlation (Fig. 1, diamonds; *R* ^2^ = 0.24), suggesting that this is a too simplistic and crude tool to use alone. Even when corrected for molecular weight (using O/C ratio divided by the molecular weight to remove effects from the size of the molecule), a poor correlation remains (Fig. 1, squares; *R* ^2^ = 0.32). Again the [O/C ratio]/molecular weight ratio is somewhat simplistic and additional factors influence water‐holding capacities beyond this simple ratio. Clearly, the oxygen atom electronegativity (3.44 Pauling units) is an important determinant in the association with water and nitrogen is also electronegative (3.04 Pauling units, i.e. less electronegative than oxygen). Using these weightings, [electronegativity/C ratio]/molecular weight provided a poorer correlation with DRH. As shown from the quantum mechanics molecular modelling (below), water molecules tend to associate primarily with oxygen atoms in our compounds, irrespective of their bonding motif. In contrast, nitrogen: water bonding is strongly affected by delocalization of the nitrogen electrons including whether it is as a primary or secondary amine or its proximity to an oxygen atom.

*α*‐hydroxyglycine has an O/C ratio of 1.5, the same as *N*‐hydroxyglycine (and, as constitutional isomers, both have a molecular weight of 91 Da). Solely considering the O/C ratio, it could be expected that they have similar DRH values. However, *N*‐hydroxyglycine has the alpha effect, due to migration of the hydroxyl (at the alpha carbon position in *α*‐hydroxyglycine) to the nitrogen in *N*‐hydroxyglycine 23. Clearly, increasing the O/C is not the only factor to consider in the water‐holding affinities for the molecules, but the location of chemical groups and their electronic influences can also impact on water binding and hence on moisturization. This is further illustrated by the DRH values from l‐homoserine and l‐threonine, which are structural isomers and so both have an O/C ratio of 0.75. Based on the O/C ratio, it was expected that both compounds would have higher DRH values than l‐serine and glycine. This was indeed the case for l‐threonine but not l‐homoserine which generated a DRH of 71.4%, that is lower than l‐serine and glycine. These results illustrate that it is not merely the atoms in a compound or the O/C ratio but the positioning/conformation of the atoms which must be considered.

Of the free linear amino acids found in NMF, serine, glycine and alanine all have DRH values between 79% and 83%. It is notable that these natural amino acids are the most abundant in NMF 9 and have the lowest DRH of all the natural amino acids tested. These natural amino acids are frequently associated with water retentive properties compared with the other natural amino acids, not only in skin but also, for example, in atmospheric aerosols and silks 24, 25. l‐serine and d‐serine provide an interesting comparison to understand the effect of chirality. As enantiomers (mirror images) have the same chemical and physical properties (other than their equal rotation of polarized light but in opposite directions and in their reactivity with other enantiomers), it was expected that chirality would not affect water‐holding capacity, and this was observed. It was also expected, and observed, that cysteine, an isostere of serine, would have a marginally higher DRH than serine because oxygen is more electronegative than sulphur. This observation highlights that replacing an atom with a less electronegative one, and consequently reducing the O/C ratio, decreases the water‐holding capacity (increases DRH).

**Figure 4:**
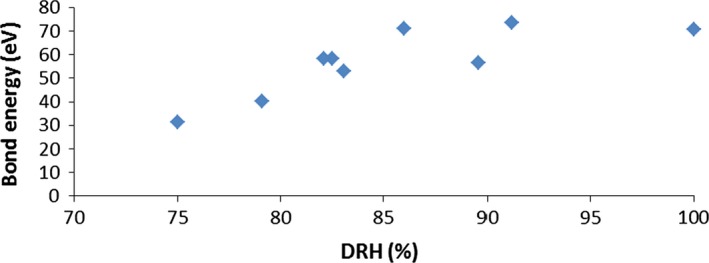
A graph showing experimental bond energy for natural amino acids (and urea) against their experimental deliquescence relative humidity (DRH) 23. From left to right: urea, glycine, d‐serine, l‐serine, l‐alanine, l‐threonine, l‐cysteine, l‐asparagine, l‐aspartic acid.

The relationship between melting point and DRH was examined because it was feasible that the higher the melting point, the greater the propensity for strong intermolecular bonding, and therefore, in solution the compound may form strong hydrogen bonds to the water, causing a lower DRH. In fact, a weak correlation (*R* ^2^ = 0.40) was found between increasing melting point and *higher* DRH values. This could be attributed to the compound forming bonds to other molecules of itself rather than to the water. Wang *et al*. showed that creating co‐crystals makes compounds less hygroscopic as hydrogen bonds form between molecules rather than to water 26. As compounds with high bond energies are typically more crystalline, they are likely to be less hygroscopic and therefore have higher DRH values. This was tested by comparing literature‐reported experimental bond energies for natural amino acids (and urea) against the DRH values 27; the data in Fig. 4 show some correlation (*R* ^2^ = 0.82) between increasing bond energies and increasing DRH, suggesting that when a compound can form strong intermolecular bonds, it is less likely to form intermolecular hydrogen bonds with water.

### Thermo‐gravimetric analysis (TGA)

The unnatural amino acids and urea were analysed by TGA following storage at 100% RH; l‐homoserine and *N*‐hydroxyglycine both deliquesced prior to TGA analysis. When heated, these materials lost ~53.3% and ~82.5% of their mass at 100°C, respectively. These values equate to 24 water molecules bound to one single *N*‐hydroxyglycine molecule and 7.5 water molecules per one l‐homoserine molecule. Considering molecular size and steric constraints, it appears implausible that 24 molecules of water could, in fact, bind directly to one molecule of *N*‐hydroxyglycine. In contrast to other hydrotropes, the TGA data from *N*‐hydroxyglycine did not distinguish between the ‘bound’ and ‘outer hydration shell’ water and so the calculated 24 bound water molecules is a significant overestimate (as shown by modelling below). *α*‐hydroxyglycine experienced a ~25.1% mass decrease between 105 and 120°C equating to 1.7 bound water molecules per *α*‐hydroxyglycine molecule. From the TGA data, it was calculated that one *N*‐hydroxyserine molecule bound to 11.5 water molecules. These results are further discussed with the QMMM findings below.

DRH was used as described above to assess the water‐scavenging ability of the compounds. The water‐holding capacities determined by DRH were validated by reference to the number of water molecules bound to each amino acid determined by TGA. Taking the results from Tables 1 and 2, the graph (Fig. 2) shows a clear trend between the quantity of water molecules and water‐holding capacity of the compounds; the lower the DRH, the greater the water‐holding capacity and scavenging ability of the compound. Again, *N*‐hydroxyglycine is an outlier for the reasons discussed above. The QMMM calculations (below) showed that only 8 water molecules associated with *N*‐hydroxyglycine which, when added to Fig. 2 as an additional data point, shows closer alignment with the DRH data confirming that DRH is a useful tool to estimate the water‐holding capacity of a compound.

### Quantum mechanics molecular modelling (QMMM)

Table 2 shows the modelled end points for the compounds evaluated by QMMM, giving the number of water molecules per compound molecule. In addition, the modelling data demonstrate the sequence and sites as water molecules attach and bind to the unnatural amino acid hydrotropes. QMMM was validated by modelling the addition of water to an established humectant, urea, and whose coordination to water has been previously published 28.

With urea, modelling showed that the first water molecule (1) hydrogen bonds to the carbonyl group, whereas the second water molecule (2) attaches to both the first water (1) and the amine. The third water molecule (3) again hydrogen bonds to the carbonyl. The fourth water (4) forms 2 hydrogen bonds directly to urea, and hence is strongly immobilized. The fifth and final water molecule (5) hydrogen bonds to the amine and to the water (3) attached to the carbonyl (for energy‐minimized structure, see Figure S2).

The literature determined that urea coordinates to 5 water molecules, with one of the waters strongly immobilized by forming 2 hydrogen bonds to urea (see Figure S3), which supports these experimental data 28. Our molecular modelling confirms that 5 water molecules coordinate, and again with one water hydrogen bonded in two places to urea. However, there are some discrepancies between the modelled coordination sites and the experimentally derived bonding sites; the QMMM suggests a more stable form of hydrated urea as the coordinated water molecules form extra hydrogen bonds between the water molecules, thereby increasing stability. The literature structure of urea has 5 waters with 6 hydrogen bonds, whereas the QMMM structure has 5 waters with 8 hydrogen bonds showing that QMMM structure of hydrated urea is more stable than that reported in the literature from NMR data.

The experimental data (DRH and TGA) indicated that *N*‐hydroxyglycine was the most hygroscopic of the unnatural linear amino acids synthesized. When modelled by QMMM, *N*‐hydroxyglycine was the only compound where the first four water molecules bind directly to the hydrotrope and where initially the first water binds to the molecule in two places, and is strongly immobilized (see Figure S4). All subsequent added water molecules bind to other bound water molecules, until the tenth to twelfth water molecules are added which then again bind to the core molecule. This may explain why the TGA data did not distinguish ‘bound’ water from ‘hydration shell’ water.

In contrast, QMMM shows that the first two water molecules bind directly, and in a similar way, to l‐homoserine, *N*‐hydroxyserine and *α*‐hydroxyglycine; the first water (1) hydrogen bonds to the hydrogen of the carboxylic acid, followed by the second water (2) hydrogen bonding to the carbonyl groups. Subsequent water molecules associate with the amino acids in different ways. For l‐homoserine, and N‐hydroxyserine, the third water molecule (3) binds between the two water molecules connected to the carboxylic acid and so is indirectly bound to the amino acid whereas in *α*‐hydroxyglycine the third water (3) forms 2 hydrogen bonds directly to *α*‐hydroxyglycine (see Figure S5). The fourth water molecule (4) hydrogen bonds to the lone pair of the alcohol group in l‐homoserine, and *N*‐hydroxyserine, whereas in *α*‐hydroxyglycine this fourth addition is to the earlier bound water molecules.

From the thermo‐gravimetric data, it was calculated that 2 water molecules bind to *α*‐hydroxyglycine, but the molecular modelling suggests that in fact, 3 water molecules would hydrogen bond to this compound. X‐ray diffractometry data indicated that *α*‐hydroxyglycine exists as a zwitterion. Therefore, the water (1) that modelling suggests binds to the hydrogen of the carboxylic acid could be precluded in the zwitterion as the hydrogen may be absent. This explanation would account for the differences observed between the TGA calculated number of water molecules bound and that predicted from QMMM.

Addition of further water molecules was modelled, and showed binding to nitrogen lone pairs and alcohol group lone pairs and to bound water molecules as described in Table 3. In essence, of the 12 water molecules coordinated to *N*‐hydroxyserine, 6 were directly hydrogen‐bonded to the amino acid with the remaining 6 bound to the attached water molecules themselves (see Figure S6).

**Table 3:** Addition sites for sequential water molecules (>5 molecules) bound to the l‐homoserine, *N*‐hydroxyserine, *N*‐hydroxyglycine and *α*‐hydroxyglycine when QMMM was performed

Water	*N*‐hydroxyserine	l‐homoserine	*N*‐hydroxyglycine	*α*‐hydroxyglycine
5	To N lone pair	To amine's H and CO lone pair	To water	To NH
6	To OH lone pair	To CO and water	To water	To N lone pair
7	To water coordinated to OH lone pair	To CO and water (inserting between last water added)	To water	To alcohol's H and water
8	To NH and water	To amine's H and CO lone pair	To water	To CO lone pair and water
9	To CO lone pair	To N lone pair and alcohol's H	To water	To 2 water molecules only
10	To water bound to CO	To NH	To N and OH lone pair	To N lone pair and water
11	To CO lone pair and 2 water molecules	To water	To CO lone pair and water	To OH lone pair and water
12	To NH and water	To 2 water molecules only	To CO lone pair and water	To 2 water molecules only

The TGA data showed that *N*‐hydroxyserine could bind 11 to 12 water molecules; this is in agreement with the modelling data. It is noteworthy that as each new water molecule was added, the hydrogen bond per water molecule ratio remained between 1.3 and 1.5. However, when the twelfth water was added, the complexes reached a higher hydrogen bond‐to‐water ratio of 1.6.

For l‐homoserine, QMMM showed that 8 water molecules could bind to this amino acid of which 5 were directly bound to l‐homoserine (see Figure S6). The TGA data also showed that 7 or 8 water molecules were bound to l‐homoserine, again in agreement with the modelling data. Indeed, the modelling shows that 8 water molecules is the optimal number to bind with l‐homoserine because, with each water molecule added, the hydrogen bond‐to‐water ratio increases from 1.3 to 1.9 up to when the eighth water was added. After the eighth water molecule, the H bond‐to‐water molecule ratio began to decrease.

### Snake skin studies

The above data show that the unnatural amino acids have a range of water‐holding capacity's and the thermal and modelling results indicate that these new unnatural amino acids can complex to 3, 8, 8 or 12 water molecules (*α*‐hydroxyglycine, l‐homoserine, *N*‐hydroxyglycine and *N*‐hydroxyserine, respectively). Efficacy of these unnatural amino acids was evaluated using snake skin, selected as a model membrane as it is dry when shed and does not contain NMF. It is axiomatic that snake skin has structural dissimilarities to human skin, with the principle lipid‐containing barrier layer (mesos layer) sandwiched between *α*‐ and *β*‐keratin layers and the presence of an external keratinized Oberhautchen. It has been shown that penetration enhancers tend to be less efficacious in snake skin studies than in human skin, probably due to reduced uptake arising from these additional barrier layers 16. However, snake skin is a good mimic of the permeability coefficient of water in human epidermal membranes 29 and is robust over 8 days and hence was selected as a biological membrane to demonstrate that the water‐holding capacities of our molecules described above do translate to an effect on a biological membrane *in vitro*.

Figure 3 shows the weight increase (assumed to be water) for snake skin samples that had been treated, blotted then dried following treatment and then exposed to 70%RH for 24 h. The percentage weight increase was calculated relative to a water‐only (control) treatment following the same protocol. The figure groups the test materials as ‘commercial preparations’ (Cetraben and Oilatum), widely used moisturizing agents (hyaluronic acid‐ HA, glycerine and urea), natural amino acids (l‐alanine to glycine), our novel hydrotropes (*α*‐hydroxyglycine to l‐homoserine) then our new materials incorporated into Oilatum cream. As expected, considering they principally hydrate human skin through occlusion, the commercial emollients (Oilatum cream and Cetraben emollient cream) did not increase the water content of snake skin whereas the widely used moisturizing agents glycerine and, to a greater extent, urea did increase water uptake into the tissue; hyaluronic acid showed no significant moisturizing effects when delivered from either 1% or 10% solutions (*P* > 0.05). The natural amino acids showed varying efficacies in our model. It is noteworthy that serine, the most abundant free amino acid in NMF and so the basis for some of our novel hydrotrope design, was the most efficacious of the natural amino acids tested.

When delivered from 10% solutions, our unnatural amino acid hydrotropes showed varying effects on water uptake into snake skin, with results correlating to the water‐scavenging results described above. So, *α*‐hydroxyglycine which was shown to be capable of binding only 3 water molecules was ineffective as a moisturizer in the snake skin study. In contrast, both *N*‐hydroxyglycine and l‐homoserine, which coordinate 8 water molecules each, significantly increased water content in the snake skin by 25% and 30%, respectively (*P* < 0.05). Unexpectedly, *N*‐hydroxyserine, shown to coordinate with 12 water molecules, showed no significant effects (*P* > 0.05) on snake skin water uptake when delivered from aqueous solution.

When applied in Oilatum, *N*‐hydroxyserine gave the greatest water uptake of all the tested systems, increasing water content over 200% when compared to a water treatment alone. Similarly, *N*‐hydroxyglycine was more potent when delivered from the commercial preparation than from water alone, increasing water uptake by 60% in our model. Both *N*‐hydroxyserine and *N*‐hydroxyglycine are highly polar molecules whereas Oilatum cream includes 6% light liquid paraffin and 15% white soft paraffin. Greater release and hence delivery of the amino acid derivatives from an oil‐containing formulation was thus expected compared with that from a simple aqueous system. Clearly, rational formulation development is merited, but the data illustrate the capacity to deliver the hydrotropes with moisturizing benefits from a cream (or oily) formulations.

As described above, the snake skin model, although useful inasmuch as NMF is absent in the tissue, does not accurately represent the structure or components of human skin 16. Snake skin contains an additional outer keratinized layer (Oberhautchen) not found in human epidermis which will consequently affect the partitioning and hence uptake of hydrotropes into snake skin, as was the case with a penetration enhancer partitioning into the membrane 16. Further, the tissue is dried prior to hydrotrope treatment which could additionally affect partitioning and uptake of these water‐soluble materials. These structural dissimilarities may explain some of the differing results with our hydrotropes, but the broad agreement between DRH, thermal and modelling data with the efficacy study illustrates the potential value of our unnatural amino acids as hydrotropes for treating dry skin. Formulation design can improve delivery beyond simply applying aqueous solutions, and clear benefits are seen in delivering the water‐soluble materials from the Oilatum cream.

## Conclusions

This study has shown that the design principles employed yielded unnatural linear amino acids with enhanced water‐holding capacities and potential efficacy as skin moisturizers.

Through the molecular design and use of reference materials yielding a library of structurally related materials, the following ‘predictive’ rules for designing hygroscopic linear amino acids can be proposed:

- Increasing the O/C ratio divided by molecular weight tends to increase the water‐holding capacity.
- Chirality has no effect on the water‐holding capacity of a compound (but can affect skin uptake).
- Compounds with high melting points and bond energies have decreased water‐holding capacity.
- When two compounds have the same [O/C ratio]/Mw, increasing the homology decreases the water‐holding capacity unless other electronic factors are involved.
- With isosteric replacement, the more electronegative atoms have the greater water‐holding capacity.

From these principles, l‐homoserine, *N*‐hydroxyglycine and *N*‐hydroxyserine were shown to have greater water‐holding capacities than their parent amino acids (serine and glycine). In comparison with a range of linear amino acids found in natural moisturizing factor and moisturizing agents such as glycerine and urea, these three chemically modified amino acids had the lowest deliquescence relative humidities and were able to bind greater numbers of water molecules as assessed by thermal analysis and molecular modelling. All three were efficacious moisturizers when used alone, but the *N*‐hydroxy‐modified structures had significantly greater effects when delivered to snake skin from Oilatum whereby *N*‐hydroxyserine increased water content by over 200% in comparison with an aqueous control treatment.

## Supporting information


**Table S1.** Analytical data used to verify successful synthesis of the compounds used in this study.
**Figure S1.** Structure of α‐hydroxyglycine determined from zero background powder X‐ray diffraction.
**Figure S2.** The energy minimised structure of urea with 5 water molecules coordinated.
**Figure S3.** The literature determination of the structure of urea with 5 water molecules coordinated 22.
**Figure S4.** Energy minimised *N*‐hydroxyglycine with water molecules. The water molecules are numbered by the order that they were added in; (A) with one hydrogen bonded water bound, (B) with 4 water molecules bound.
**Figure S5.** Energy minimised unnatural amino acids with water molecules. The water molecules are numbered by the order that they were added in; (A) l‐homoserine with four water molecules bound. (B) *N*‐hydroxyserine with 4 water molecule bound. (C) α‐hydroxyglycine with three water molecules bound.
**Figure S6.** (A) Energy minimised structure of *N*‐hydroxyserine with 12 water molecules hydrogen bonded. *N*‐hydroxyserine with 12 water molecule bound; (B) The energy minimised structure of l‐homoserine with 8 water molecules hydrogen bonded.

## References

[1] Williams, A.C. Transdermal and Topical Drug Delivery. Pharmaceutical Press, London (2003).

[2] Nakagawa, N. , Sakai, S. , Matsumoto, M. and Yamada, K. Relationship between NMF (lactate and potassium) content and the physical properties of the stratum corneum in healthy subjects. J. Invest. Dermatol. 122, 755–763 (2004). 15086563 10.1111/j.0022-202X.2004.22317.x

[3] O'Regan, G.M. , Kemperman, P.M.J.H. , Sandilands, A. *et al* Raman profiles of the stratum corneum define 3 filaggrin genotype‐determined atopic dermatitis endophenotypes. J. Allergy Clin. Immunol. 126, 574–580 (2010). 20621340 10.1016/j.jaci.2010.04.038 PMC3627961

[4] Fluhr, J.W. , Darlenski, R. and Surber, C. Glycerol and the skin: holistic approach to its origin and functions. Brit. J. Dermatol. 159, 23–34 (2008). 18510666 10.1111/j.1365-2133.2008.08643.x

[5] McGrath, J.A. Filaggrin and the great epidermal barrier grief. Australasian J. Dermatol. 49, 67–74 (2008). 10.1111/j.1440-0960.2008.00443.x 18412804

[6] Cork, M.J. , Danby, S.G. , Vasilopoulos, Y. , Hadgraft, J.A. , Lane, M.E. and Moustafa, M. Epidermal barrier dysfunction in atopic dermatitis. J. Invest. Dermatol. 129, 1892–1908 (2009). 19494826 10.1038/jid.2009.133

[7] Jokura, Y. , Ishikawa, S. , Tokuda, H. and Imokawa, G. Molecular analysis of elastic properties of the stratum corneum by solid state ^13^C‐nuclear magnetic resonance spectroscopy. J. Invest. Dermatol. 104, 806–812 (1995). 7537776 10.1111/1523-1747.ep12607005

[8] Caspers, P.J. , Lucassen, G.W. , Carter, E.A. , Bruining, H.A. and Puppels, G.J. *In vivo* confocal Raman microspectroscopy of the skin: noninvasive determination of molecular concentration profiles. J. Invest. Dermatol. 116, 434–442 (2001). 11231318 10.1046/j.1523-1747.2001.01258.x

[9] Burke, R.C. Free amino acids and water soluble peptides in stratum corneum and skin surface film in human beings. Yale J. Biol. Med. 38, 355–373 (1996). PMC2591189 5950260

[10] Koyama, J. , Horii, I. , Kawasaki, K. , Nakayama, Y. , Morikawa, Y. and Mitsui, T. Free amino acids of stratum corneum as a biochemical marker to evaluate dry skin. J. Soc. Cosmet. Chem. 35, 183–195 (1984).

[11] Visscher, M. , Robinson, M. and Wickett, R.R. Regional variation in the free amino acids in the stratum corneum. J. Cosmet. Sci. 61, 303–309 (2010). 20716438

[12] Egawa, M. and Tagami, H. Comparison of the depth profiles of water and water‐binding substances in the stratum corneum determined *in vivo* by Raman spectroscopy between the cheek and volar forearm skin: effects of age, seasonal changes and artificial forced hydration. Br. J. Dermatol. 158, 251–260 (2008). 18047517 10.1111/j.1365-2133.2007.08311.x

[13] Robinson, M. , Visscher, M. , LaRuffa, A. and Wickett, R.R. Natural moisturising factors (NMF) in the stratum corneum (SC). II. Regeneration of NMF over time after soaking. J. Cosmet. Sci. 61, 23–29 (2010). 20211114

[14] Sasaki, O. , Kanai, I. and Yazawa, Y. Relationship between the chemical structure of humic substances and their hygroscopic properties. Science 1, 17–22 (2007).

[15] Veguilla, R . Temperature dependence on the deliquescence relative humidity of inorganic aerosol particles. University of Harvard. Available at: http://88.198.249.35/preview/S9ka-rOHn1GRCZApFVrZarbmCdr0TOxDPLHLobZYGUw,/Temperature-Dependence-on-the-Deliquescence-Relative.html?query=Relative-Humidity-Graph, accessed 2015 January 15.

[16] Rigg, P.C. and Barry, B.W. Shed snake skin and hairless mouse skin as model membranes for human skin during permeation studies. J. Invest. Dermatol. 94, 235–240 (1990). 2299198 10.1111/1523-1747.ep12874561

[17] O'Brien, F.E.M. The control of humidity by saturated salt solutions. J. Sci. Instrum. 25, 73–76 (1948).

[18] Hoefnagel, A.J. , Bekkum, H.V. and Peters, J.A. The reaction of glyoxylic acid with ammonia revisited. J. Org. Chem. 57, 3916–3921 (1992).

[19] Buehler, E. and Brown, G.B. A general synthesis of *N*‐hydroxyamino acids. J. Org. Chem. 32, 265–267 (1967).

[20] Goto, G. , Kawakita, K. , Okutani, T. and Miki, T. An improved synthesis of *N*‐hydroxyamino acids and their esters using (Z)‐2‐furaldehyde oxime. Chem. Pharm. Bull. 34, 3202–3207 (1986).

[21] Flynn, T.C. , Petros, J. , Clark, R.E. and Vieham, G.E. Dry skin and moisturisers. Clin. Dermatol. 19, 387–392 (2001). 11535378 10.1016/s0738-081x(01)00199-7

[22] Weber, T.C. , Kausch, M. , Rippke, F. , Schoelermann, A.M. and Filbry, A.W. Treatment of xerosis with a topical formulation containing glyceryl glucoside, natural moisturizing factors, and ceramide. J. Clin. Aesthet. Dermatol. 5, 29–39 (2015). PMC3424590 22916312

[23] Childress, S. Alpha‐effect in flux ropes and sheets. Phys. Earth Planet. Inter. 20, 172–180 (1979).

[24] Chan, M.N. , Choi, M.Y. , Ng, N.L. and Chan, C.K. Hygroscopicity of water‐soluble organic compounds in atmospheric aerosols: amino acids and biomass burning derived organic species. Environ. Sci. Technol. 39, 1555–1562 (2005). 15819209 10.1021/es049584l

[25] Sezutsu, H. , Kajiwara, H. , Kojima, K. , Mita, K. , Tamura, T. , Tamada, Y. and Kameda, T. Identification of four major hornet silk genes with a complex of alanine‐rich and serine‐rich sequences in *Vespa simillima xanthoptera* cameron. Biosci. Biotechnol. Biochem. 71, 2725–2734 (2001). 10.1271/bbb.70326 17986776

[26] Wang, Z.Z. , Chen, J.M. and Lu, T.B. Enhancing the hygroscopic stability of S‐oxiracetam via pharmaceutical cocrystals. Cryst. Growth Des. 12, 4562–4566 (2012).

[27] The experimental bond energies an article containing tables summarizing the results of the calculated experimental parameters of 800 exemplary solved molecules found in The Grand Theory of Classical Physics. Available at: http://www.blacklightpower.com/theory/bookdownload.shtml, accessed 2015 January 15.

[28] Rezus, Y.L.A. and Bakker, H.J. Effect of urea on the structural dynamics of water. Proc. Natl Acad. Sci. USA 103, 18417–18420 (2006). 17116864 10.1073/pnas.0606538103 PMC1693679

[29] Itoh, T. , Xia, J. , Magavi, R. , Nishihata, T. and Rytting, J.H. Use of shed snake skin as a model membrane for *in vitro* percutaneous penetration studies: comparison with human skin. Pharm. Res. 7, 1042–1047 (1990). 2281034 10.1023/a:1015943200982

