# Supplementary material for: Design, synthesis and characterization of linear unnatural amino acids for skin moisturization
Source: Int J Cosmet Sci. 2016 Jul 24;39(1):72–82. doi: 10.1111/ics.12351 (PMC5244679; doi:10.1111/ics.12351)

**Supporting information**

**CONTENTS:**

Supporting information Table 1: Analytical data used to verify successful synthesis of the compounds used in this study

Supporting information Figure 1: Structure of α-hydroxyglycine determined from zero background powder X-ray diffraction.

Supporting information Figure 2: The energy minimised structure of urea with 5 water molecules coordinated.

Supporting information Figure 3: The literature determination of the structure of urea with 5 water molecules coordinated [22].

Supporting information Figure 4: Energy minimised *N*-hydroxyglycine with water molecules. The water molecules are numbered by the order that they were added in; A) with one hydrogen bonded water bound, B) with 4 water molecules bound.

Supporting information Figure 5: Energy minimised unnatural amino acids with water molecules. The water molecules are numbered by the order that they were added in; A) L-homoserine with four water molecules bound. B) *N*-hydroxyserine with 4 water molecule bound. C) α-hydroxyglycine with three water molecules bound.

Supporting information Figure 6: A) Energy minimised structure of *N*-hydroxyserine with 12 water molecules hydrogen bonded. *N*-hydroxyserine with 12 water molecule bound; B) The energy minimised structure of L-homoserine with 8 water molecules hydrogen bonded.

**Supporting information Table 1**: Analytical data used to verify successful synthesis of the compounds used in this study

| **Chemical** | **Structure** | **δ_H_ (400 MHz, CDCl_3_)**  **ppm** | **δC (100 MHz, CDCl_3_)**  **ppm** | **ν_max_ (FT IR, CHCl3, KBr plates)/ cm^-1^** | **m/z** | **Melting point**  **°C** |
| --- | --- | --- | --- | --- | --- | --- |
| Benzaldoxime |  | 7.38 (2H, m, H-5, H-3)  7.58 (2H, m, H-2, H-6)  7.88 (1H, t, J=1.4Hz, H-4)  8.15 (1H, s, H-7) | 127.0 (C-5 and C-3)  128.8 (C-6 and C-2)  130.0 (C-4)  131.8 (C-1)  150.4 (C-7) | 3280, 3061 (C-H), 3028 (C-H), 2985 (C-H), 2898, 2358, 2341, 1896, 1810, 1697 (C=N), 1631 (N-H), 1598 (C=C), 1577 (C=C), 1492 (N-O), 1443, 1303, 1288, 1209, 1176, 1158, 1102, 1074, 946, 868, 752, 702, 644. | 122.0597 ([M+H]^+^, 100%) by FTMS + ESI  C_7_H_­8_NO requires 122.0600 | liquid |
| 2-bromo-3-hydroxypropanoic acid |  | 3.97-4.09 (2H, ddd, J=5.2Hz, J’=12.1Hz, J’’=25.8Hz, H-2)  4.40 (1H, t, J=5.3Hz, H-3) | 43.8 (C-2)  63.7 (C-3)  172.8 (C-4) | 3407 (O-H), 2931 (C-H), 2355, 1722, 1615 (C=O), 1452, 1398, 1290, 1269, 1245, 1191 (C-O), 1160 (C-O), 1068, 1026, 909 | 166.9 [30 %, (M-H)^+^] by LCMS  C_3_H_5_O_3_Br requires 166.9394 |  |
| Ethyl 2-bromo-3-hydroxypropanoate |  | 1.30 (3H, t, J=6.8Hz, H-6)  2.41 (1H, br s, H-1) 3.92-4.07 (2H, ddd, J=7.6Hz, J’=12.0Hz, J’’=40.0Hz, H-2)  4.25 (2H, J=7.2Hz, H-5)  4.30 (1H, t, J=1.6Hz, H-3) | 13.92 (C-6)  44.61 (C-3)  62.43 (C-5)  63.85 (C-2)  166.7 (C-4) | 3434 (O-H), 2987 (C-H), 2939 (C-H), 1736 (C=O), 1464, 1373, 1298, 1269, 1244, 1184, 1152, 1098 (C-O), 1080 (C-O), 1041 (C-O), 951, 857, 797, 678, 615 |  |  |
| *N*-hydroxyglycine |  | 1.18 (1H, br s, N-H)  3.62 (2H, br s, H-1) | 55.23 (C-1)  174.69 (C-2) | 3375, 3234, 3094 (O-H, N-H), 2908, 1645 (C=O), 1592, 1549, 1508, 1406, 1305, 1214, 1178. | 116.9 [100 %] by LCMS  C_2_H_5_O_3_N requires 91.0269 | 137-139 |
| *N*-hydroxyserine |  | 3.12 (1H, m, H-8)  3.56 (1H, m, H-8)  4.59 (1H, m, H-3) |  | 3308 (O-H), 3032 (COOH), 2908 (CH), 1648 (N-H), 1591 (C=O), 1540 (N-O), 1511, 1402, 1306, 1229, 1174 | 141.0 ([M]^+^, 100 %) by FTMS + ESI  C_3_H_­7_NO_4_ requires 121.0735 | 159-163 |
| α-hydroxyglycine |  | 4.93 (1H, s, H-1) | 26.81 (C-1)  175.83 (C-2) | 3056 (O-H, C-H), 1637 (C=O), 1561 (N-H), 1449, 1394, 1352, 1305 (C-O), 1194, 1129, 1071 (C-N), 881, 827, 616, 564, 536. | 169.0228 [(M+Na)^-^, 100 %] by FTMS + ESI  C_4_H_6_N_2_Na O_4_^-^ requires 169.0231  C_2_H_5_O_3_N requires 91.0269 | 105 - 108^o^C (dec.) |

**Supporting information Figure 1:** Structure of α-hydroxyglycine determined from zero background powder X-ray diffraction.


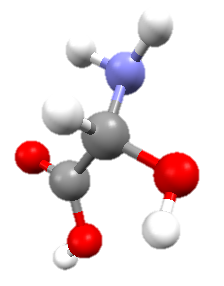


**Supporting information Figure 2**: The energy minimised structure of urea with 5 water molecules coordinated.


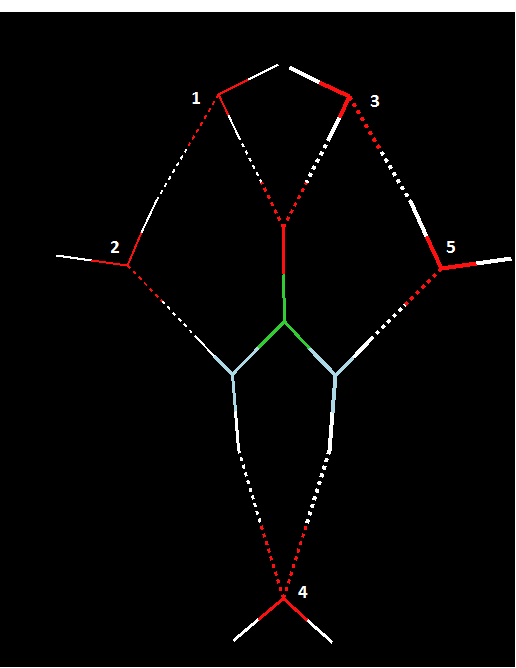


Supporting information Figure 3: The literature determination of the structure of urea with 5 water molecules coordinated [22].

Supporting information Figure 4: Energy minimised *N*-hydroxyglycine with water molecules. The water molecules are numbered by the order that they were added in; A) with one hydrogen bonded water bound, B) with 4 water molecules bound.


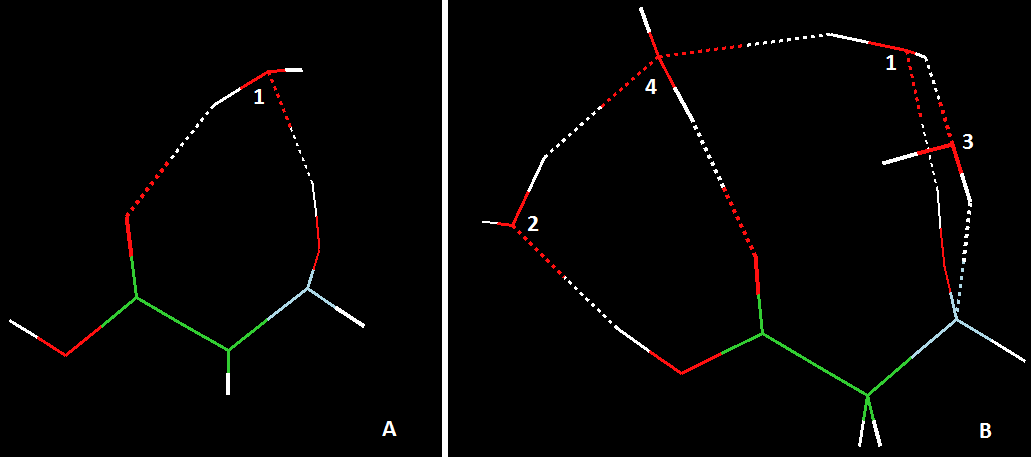


**Supporting information Figure 5**: Energy minimised unnatural amino acids with water molecules. The water molecules are numbered by the order that they were added in; A) L-homoserine with four water molecules bound. B) *N*-hydroxyserine with 4 water molecule bound. C) α-hydroxyglycine with three water molecules bound.


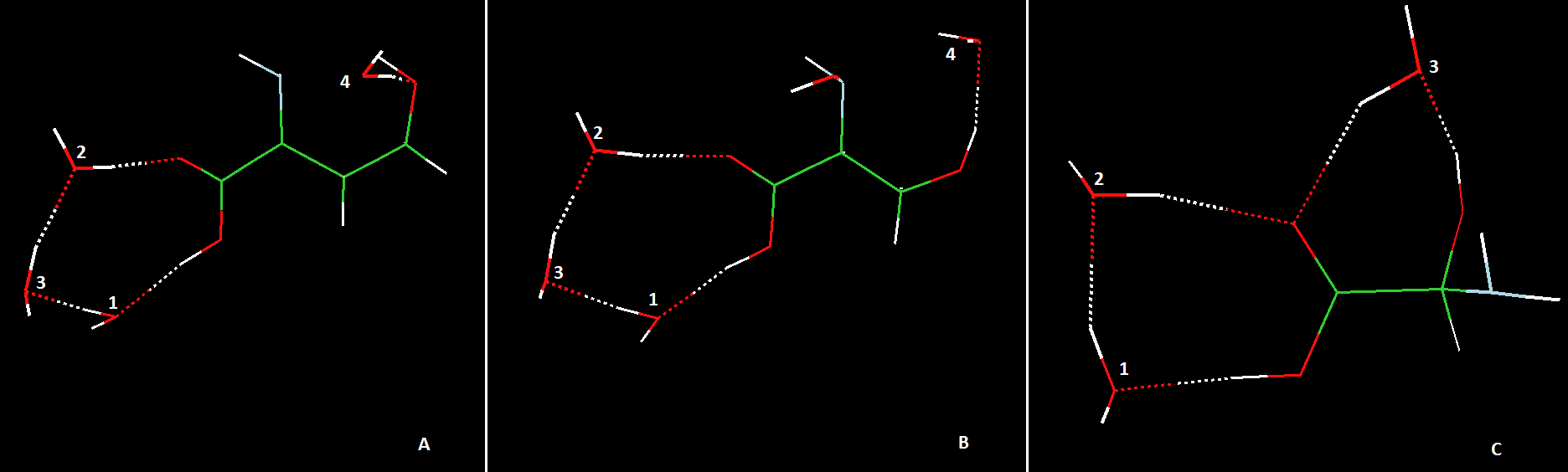


**Supporting information Figure 6**: A) Energy minimised structure of *N*-hydroxyserine with 12 water molecules hydrogen bonded. *N*-hydroxyserine with 12 water molecule bound; B) The energy minimised structure of L-homoserine with 8 water molecules hydrogen bonded.


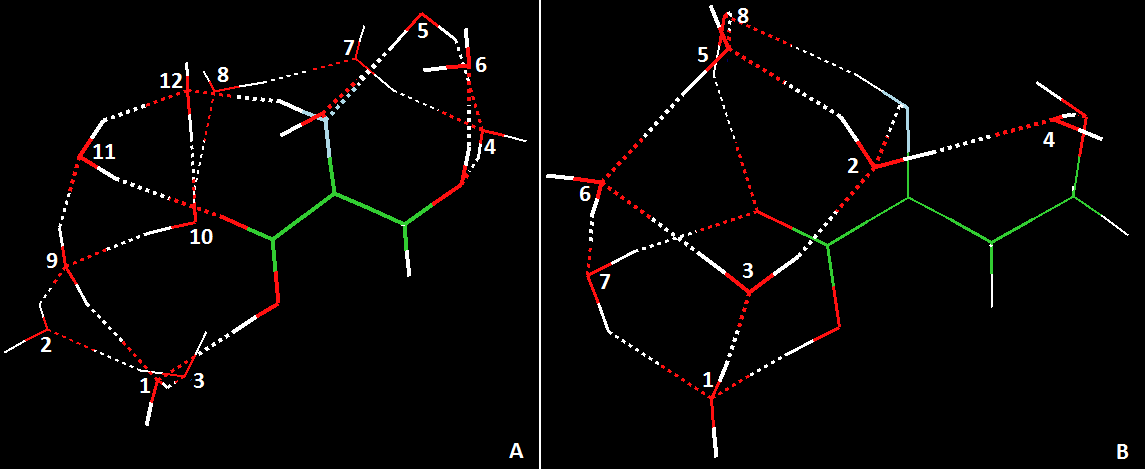

Supplement: Supplementary file 1 — Table S1. Analytical data used to verify successful synthesis of the compounds used in this study. Figure S1. Structure of α‐hydroxyglycine determined from zero background powder X‐ray diffraction. Figure S2. The energy minimised structure of urea with 5 water molecules coordinated. Figure S3. The literature determination of the structure of urea with 5 water molecules coordinated 22. Figure S4. Energy minimised N‐hydroxyglycine with water molecules. The water molecules are numbered by the order that they were added in; (A) with one hydrogen bonded water bound, (B) with 4 water molecules bound. Figure S5. Energy minimised unnatural amino acids with water molecules. The water molecules are numbered by the order that they were added in; (A) l‐homoserine with four water molecules bound. (B) N‐hydroxyserine with 4 water molecule bound. (C) α‐hydroxyglycine with three water molecules bound. Figure S6. (A) Energy minimised structure of N‐hydroxyserine with 12 water molecules hydrogen bonded. N‐hydroxyserine with 12 water molecule bound; (B) The energy minimised structure of l‐homoserine with 8 water molecules hydrogen bonded. [file ICS-39-72-s001.docx]
